# Using de novo transcriptomes to decipher the relationships in cutthroat trout subspecies (*Oncorhynchus clarkii*)

**DOI:** 10.1111/eva.13735

**Published:** 2024-07-11

**Authors:** Andrea L. Kokkonen, Peter C. Searle, Dennis K. Shiozawa, R. Paul Evans

**Affiliations:** ^1^ Department of Microbiology and Molecular Biology Brigham Young University Provo Utah USA; ^2^ Department of Ecology and Evolutionary Biology Cornell University Ithaca New York USA; ^3^ Department of Biology Brigham Young University Provo Utah USA

**Keywords:** cutthroat trout, *Oncorhynchus clarkii*, transcriptomes

## Abstract

For almost 200 years, the taxonomy of cutthroat trout (*Oncorhynchus clarkii*), a salmonid native to Western North America, has been in flux as ichthyologists and fisheries biologists have tried to describe the diversity within these fishes. Starting in the 1950s, Robert Behnke reexamined the cutthroat trout and identified 14 subspecies based on morphological traits, Pleistocene events, and modern geographic ranges. His designations became instrumental in recognizing and preserving the remaining diversity of cutthroat trout. Over time, molecular techniques (i.e. karyotypes, allozymes, mitochondrial DNA, SNPs, and microsatellite arrays) have largely reinforced Behnke's phylogenies, but have also revealed that some relationships are consistently weakly supported. To further resolve these relationships, we generated de novo transcriptomes for nine cutthroat subspecies, as well as a Bear River Bonneville form and two Colorado River lineages (blue and green). We present phylogenies of these subspecies generated from multiple sets of orthologous genes extracted from our transcriptomes. We confirm many of the relationships identified in previous morphological and molecular studies, as well as discuss the importance of significant differences apparent in our phylogenies from these studies within a geological perspective. Specific findings include three distinct clades: (1) Bear River Bonneville form and Yellowstone cutthroat trout; (2) Bonneville cutthroat trout (*n* = 2); and (3) Greenback and Rio Grande cutthroat trout. We also identify potential gene transfer between Bonneville cutthroat trout and a population of Colorado River green lineage cutthroat trout. Using these findings, it appears that additional groups warrant species‐level consideration if other recent species elevations are retained.

## 1 INTRODUCTION

**FIGURE 1:**
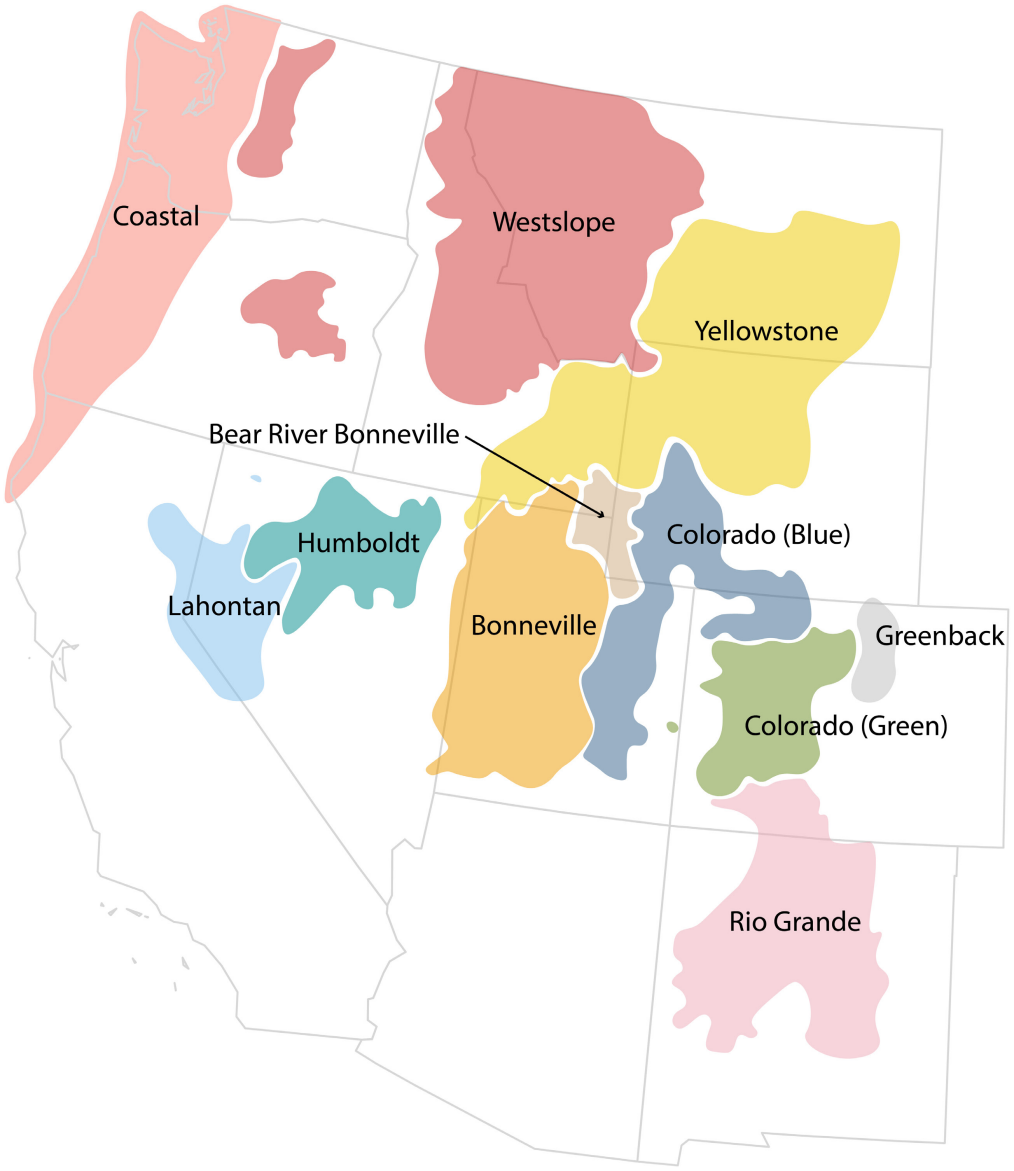
Map of ranges of cutthroat trout subspecies, lineages, and forms used in this study. “Free United States SVG Map” used with permission from simplemaps.com. Ranges adapted from literature, as well as www.nativetroutflyfishing.com.

Cutthroat trout (*Oncorhynchus clarkii*) have been separated into a varying number of subspecies (Table 1). Their current range spans from Alaska to New Mexico (Figure 1) and they are found in diverse habitats from coastal temperate rainforests to desert streams (Loxterman & Keeley, 2012). The subspecies tend to be endemic to major drainage basins, reflecting both current and historic drainage connections and isolation events. Through these dynamic processes, cutthroat trout underwent local adaptation and genetic drift, eventually giving rise to a range of morphological variation between subspecies (Allendorf & Leary, 1988; Link & Keeley, 2018). While variation can lead to adaptability and survival (Carneiro & Lyko, 2020; Kardos et al., 2021), it has also led to confusion in both taxonomic classification and fisheries management (Behnke, 1988, 1992; Gall & Loudenslager, 1981; Loudenslager & Gall, 1980; Loudenslager & Thorgaard, 1979; Loxterman & Keeley, 2012; Martin et al., 1985; Pritchard et al., 2009; Shiozawa et al., 2010, 2018; Shiozawa & Williams, 1992; Utter & Allendorf, 1994; Wilson & Turner, 2009).

**TABLE 1:** Cutthroat subspecies prior to the recent recommendation to elevate four subspecies.

Common name	Scientific name	Status
Coastal	*O. clarkii clarkii* ^1^	Accepted
Westslope	*O. clarkii lewisi* ^2^	Accepted, 9 lineages hypothesized
Alvord^a^	*O. clarkii alvordensis* ^3^	Accepted
Humboldt	*O. clarkii humboldtensis* ^3^	Accepted
Lahontan	*O. clarkii henshawi* ^3^	Accepted, 2 lineages hypothesized
Paiute	*O. clarkii seleniris* ^3^	Accepted
Bonneville	*O. clarkii utah* ^4^	Accepted, Bear River form hypothesized
Colorado River	*O. clarkii pleuriticus* ^4^	Accepted, 3 lineages hypothesized
Greenback	*O. clarkii stomias* ^4^	Accepted, subject to revalidation
Rio Grande	*O. clarkii virginalis* ^4^	Accepted
Snake River fine spotted	*O. clarkii behnkei* ^4^	Debated
Yellowfin^a^	*O. clarkii macdonaldi* ^4^	Accepted
Yellowstone	*O. clarkii bouvieri* ^4^	Accepted

*Note*: Forms indicate that the relationships are not phylogenetic while lineages suggest that the relationships share common ancestry.

^a^ Extinct. Numerical superscripts identify the four species names proposed by the Committee on Names of Fishes (Page et al., 2023). ^1^ *O. clarkii*, ^2^ *O. lewisi*, ^3^ *O. henshawi*, ^4^ *O. virginalis*.

The earliest attempt to classify these subspecies was made by George Suckley (1874), who delineated these and other trout species using ecological similarities rather than with the evolutionary concepts now used in phylogenetic studies. In the following years, taxonomists focused on morphological and meristic data to differentiate subspecies (Bestgen et al., 2019; Jordan, 1891, 1920), but because these morphometric traits regularly overlapped between the subspecies, the cutthroat trout were often inconsistently classified. Early management practices also impacted the delineation of cutthroat trout subspecies. Confronted with reductions or loss of cutthroat trout fisheries, managers focused on re‐establishing the depleted fisheries with fish from alternative sources, rather than reviving local populations (Behnke, 2002; Dunham et al., 1997; Metcalf et al., 2012; Young, 1995). Initially, rainbow trout were stocked to bolster populations and reestablish fisheries (Shiozawa et al., 2018). However, rainbow trout readily hybridize with cutthroat trout (Allendorf & Leary, 1988; Kovach et al., 2015; Meyer et al., 2017), so these introductions led to the formation of many introgressed populations. Cutthroat trout were also used in place of rainbow trout to supplement native cutthroat trout populations or to establish new populations in headwater ecosystems (Dunham et al., 1997). Relocating cutthroat trout from one area to another was readily practiced, leading to the introduction of nonnative cutthroat trout throughout Western United States (Metcalf et al., 2012; Miller, 1950; Shiozawa et al., 2018). These introductions, based on limited data and an underappreciation for the preservation of local native populations (Bahls, 1992), ultimately led to widespread hybridization between distinct lineages.

Robert Behnke (1979, 1988, 1992, 2002) re‐emphasized cutthroat trout diversity, identifying 14 subspecies based on morphological and meristic comparisons, coloration, spotting patterns, and known historical geographical distribution (Bestgen et al., 2019; Shiozawa et al., 2018). This documented the inherent geographical diversity of cutthroat trout subspecies and ultimately proved invaluable for their preservation. However, the morphological and meristic traits he used, such as mean counts of basibranchial teeth, pyloric caeca, lateral line scales, and spotting patterns, often overlap between the subspecies, making it difficult to confidently distinguish one from another (Shiozawa et al., 2018).

In the mid‐to‐late 20th century, emerging molecular techniques provided an alternative approach to evaluate the relationships between the cutthroat trout subspecies. Karyotypes and allozymes offered further insight into the subspecies (Gyllensten et al., 1985). For example, allozymes suggested that the trout in the Bear River subbasin of the Bonneville Basin were more closely related to the Yellowstone cutthroat trout than the Bonneville cutthroat trout in the remainder of the Bonneville Basin, suggesting that the Bonneville cutthroat trout subspecies was paraphyletic, with two forms rather than two monophyletic lineages that shared a direct common ancestor (Loudenslager & Gall, 1980; Martin et al., 1985). Eventually, molecular studies began to focus on mitochondrial DNA (mtDNA; Bestgen et al., 2019; Loxterman & Keeley, 2012; Metcalf et al., 2007, 2012; Pritchard et al., 2009; Shiozawa et al., 2010; Smith et al., 2002; Wilson & Turner, 2009). Initially, restriction enzyme digests of mtDNA were used to clarify the interpretation of genetic lineages. While the mtDNA restriction fragment length polymorphisms (RFLPs) led to well‐supported relationships for some of the subspecies (Pritchard et al., 2009; Shiozawa et al., 2010; Wilson & Turner, 2009), they did not successfully define all relationships, especially those found in the interior west (Yellowstone complex; Bear River Bonneville form, Bonneville, Colorado River, Greenback, Rio Grande, and Yellowstone cutthroat trout). Other studies using a limited number of mitochondrial genes (Bestgen et al., 2019; Metcalf et al., 2012) were also unable to resolve all relationships in the Yellowstone complex.

**FIGURE 2:**
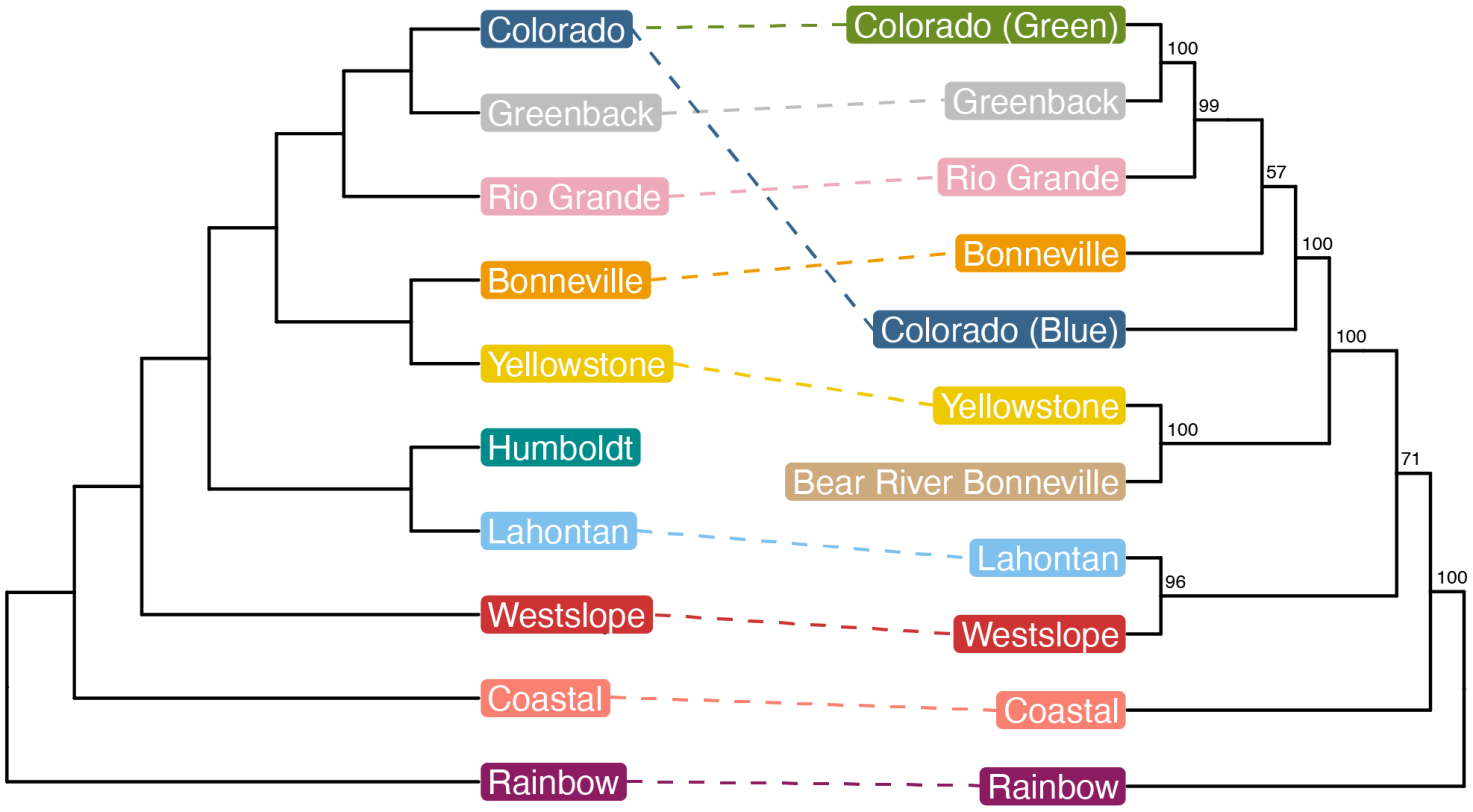
Comparison of trees generated by Robert Behnke (2002; left) and Shiozawa et al. (2018; right). Only subspecies, lineages, and forms used in this project are included. Colorado (blue) is Colorado River blue lineage and Colorado (green) is Colorado River green lineage.

Building on this work with mtDNA, Shiozawa et al. (2018) sequenced nine mitochondrial genes. They found that the most ancestral subspecies (Coastal, Westslope, and Lahontan cutthroat trout) had relationships consistent with Behnke's phylogeny (Figure 2). They also found additional support for a close relationship between Yellowstone and Bonneville cutthroat trout in the Bear River subbasin (Bear River Bonneville form), which supports part of Behnke's hypothesis that Bonneville cutthroat trout in the Bear River subbasin originated from the late Pleistocene transfer of the Bear River into the Bonneville Basin (Behnke, 1981). In contrast to the close relationship between Yellowstone cutthroat trout and the Bear River Bonneville form, they also showed that cutthroat trout native to the main Bonneville Basin were more closely related to the remaining Southern Rocky Mountain–Bonneville cutthroat trout rather than to Yellowstone cutthroat trout. This suggests that the main basin Bonneville cutthroat trout did not originate from Yellowstone cutthroat trout entering the Bonneville Basin with the Bear River capture event, though the relationship between Bonneville cutthroat trout and the remaining Southern Rocky Mountain–Bonneville cutthroat trout subspecies had the lowest bootstrap value in their phylogeny.

To date, studies using nuclear markers with multiple cutthroat trout subspecies are limited. One study using microsatellites looked at a small subset of cutthroat trout (Colorado River, Greenback, Rio Grande, and Yellowstone) and found 12 tetranucleotide microsatellite loci that were polymorphic in all four subspecies but lacked fixed diagnostic differences (Pritchard et al., 2007). Following up on this study, Pritchard et al. (2012) found that their single nucleotide polymorphisms (SNPs) and SNP‐type diagnostic markers were identical for Bonneville, Colorado River, Greenback, Rio Grande, and Yellowstone cutthroat trout and thus, referred to the group collectively as the Yellowstone group. Additionally, Campbell et al. (2012) identified 200 SNPs that successfully differentiated five cutthroat trout subspecies (Bonneville, Coastal, Lahontan, Westslope, and Yellowstone cutthroat trout). Using nine subspecies, Houston et al. (2012) identified 125 SNP loci that differentiated most of the major subspecies from each other. However, these SNPs were unable to resolve several subspecies in the Yellowstone complex (specifically Bear River Bonneville form/Yellowstone and Colorado River/Greenback cutthroat trout). The Greenback cutthroat trout specimens used in this study were later determined to be Colorado River cutthroat trout that were stocked outside their native range (Metcalf et al., 2012), which could explain the lack of resolution between these subspecies. Additional studies using SNPs to evaluate genetic variation between subspecies focused on a limited number of cutthroat trout subspecies (Kalinowski et al., 2011; Pritchard et al., 2013).

All previous studies have demonstrated the complexity of reconstructing cutthroat trout lineages—a task that has spanned decades and remains incompletely resolved today. Although the phylogenetic studies have progressively presented strong support for many of the relationships among subspecies, there is still much debate regarding relationships within the Southern Rocky Mountain–Bonneville complex. Recent taxonomic revisions to the cutthroat trout have been recommended by the American Fisheries Society & American Society of Ichthyologists & Herpetologists (Page et al., 2023) as suggested by Markle (2018). Following these recommendations, the cutthroat trout would be split into four species (Coastal, Westslope, Lahontan, and Rocky Mountain cutthroat trout), some of which would contain multiple subspecies. These taxonomic designations have been debated (Trotter et al., 2018) as they have implications for management of these subspecies, and thus, we use the original designations as outlined by Behnke (2002) in this study. Before elevating these subspecies, additional research is needed to fully delineate cutthroat trout, specifically the aforementioned Rocky Mountain cutthroat trout, which would consist of five major cutthroat trout subspecies as recognized by Behnke (2002). Although this group has been difficult to resolve, nuclear data have not been extensively evaluated, despite the utility for species delimitation.

In this paper, we aim to further resolve the phylogenetic relationships between cutthroat trout, particularly the Southern Rocky Mountain–Bonneville complex, using de novo transcriptomes. We chose to use transcriptomes because they are a good approximation of phylogenomics at a reduced sequencing and bioinformatic cost (Cheon et al., 2020). We assembled transcriptomes for nine subspecies as well as two lineages (Colorado River blue and green lineages; Metcalf et al., 2007, 2012) and one form (Bear River Bonneville form). Using sets of orthologous genes extracted from these transcriptomes, we evaluate the phylogenetic relationships of these subspecies. We confirm many of the relationships identified in previous studies as well as discuss significant differences in our phylogeny from these studies using a geological perspective. Additionally, we highlight the diversity found within the Rocky Mountain cutthroat trout—as described by Markle (2018)—and discuss the implications this has on potential taxonomic revisions of cutthroat trout.

## 2 METHODS

### 2.1 Specimen collection

Tissues were collected from nine cutthroat trout subspecies between 2012 and 2022 with the help of state biologists and local fishermen (IACUC‐approved protocol #15‐0602; Table 2). A total of 14 specimens were collected, including Bonneville (main basin Bonneville form *n* = 2; Bear River Bonneville form *n* = 1), Coastal (*n* = 1), Colorado River (blue lineage *n* = 2; green lineage *n* = 2), Greenback (*n* = 1), Humboldt (*n* = 1), Lahontan (*n* = 1), Rio Grande (*n* = 1), Westslope (*n* = 1), and Yellowstone cutthroat trout (*n* = 1). For this study, we distinguish between forms, which are relationships within the same subspecies that are paraphyletic, and lineages, which represent relationships within a subspecies that share a common ancestor (monophyletic).

**TABLE 2:** Collection data of cutthroat trout subspecies, lineages, and forms used in this study.

Common name	Species	Year	Location
Bear River Bonneville form	*O. clarkii utah*	2012	Big Creek, UT
Bonneville	*O. clarkii utah*	2022	Big Wash Creek, NV
Bonneville	*O. clarkii utah*	2012	Diamond Fork River, UT
Coastal	*O. clarkii clarkii*	2019	Mack Creek, OR
Colorado River blue lineage	*O. clarkii pleuriticus*	2022	Little West Fork of Blacks Fork, UT
Colorado River blue lineage	*O. clarkii pleuriticus*	2022	Right Fork UM Creek, UT
Colorado River green lineage	*O. clarkii pleuriticus*	2022	Bobtail Creek, CO
Colorado River green lineage	*O. clarkii pleuriticus*	2022	Beaver Creek, UT
Greenback	*O. clarkii stomias*	2018	Bear Creek, CO
Humboldt	*O. clarkii humboldtensis*	2012	Mary's River, NV
Lahontan	*O. clarkii henshawi*	2016	Bettridge Creek, UT
Rio Grande	*O. clarkii virginalis*	2021	Hay Press Lake, CO
Westslope	*O. clarkii lewisi*	2019	Middle Fork of Salmon River, ID
Yellowstone	*O. clarkii bouvieri*	2019	Soldier Creek, WY

Fish were euthanized using a mixture of sodium bicarbonate, water, and MS‐222 (MilliporeSigma, St. Louis, MO, USA) for ~15 min. Heart, gill, muscle, liver, and eyes were removed, thinly sliced, and stored in RNAlater (MilliporeSigma, St. Louis, MO, USA) to stop the breakdown of RNA by RNase. These tissues were flash frozen in liquid nitrogen, transported on dry ice to Brigham Young University, and stored at −80°C until processing. Tissues were deposited into the Monte L. Bean Life Science Museum, Provo, UT.

### 2.2 Sequencing

Tissues from 14 specimens (muscle *n* = 14; heart *n* = 1) were sequenced either using short‐read Illumina RNA sequencing or long‐read PacBio RNA sequencing. Short‐read sequencing was completed at Brigham Young University's DNA Sequencing Center (Provo, UT, USA) and Novogene America (Davis, CA, USA). For nine specimens, total RNA was extracted from muscle tissue using TRIzol Reagent (Thermo Fisher Scientific, Waltham, MA, USA) according to the manufacturer's instructions. RNA degradation and contamination were monitored on 1% agarose gels. RNA quality and purity were checked by an Agilent 2100 Bioanalyzer (Santa Clara, CA, USA) and a NanoDrop (Thermo Fisher Scientific, Waltham, MA, USA). The isolation of mRNA, via rRNA depletion, and construction of cDNA libraries were performed by the BYU DNA Sequencing Center or Novogene America for Illumina paired‐end sequencing using the Illumina TruSeq RNA Sample Prep Kit. All libraries were sequenced on an Illumina HiSeq 2000 platform (Illumina, San Diego, CA, USA) with paired‐end reads (Table S1).

We sequenced five specimens at Brigham Young University's DNA Sequencing Center using long‐read sequencing in 2021 and 2022 (Table S2). The same tissue was sequenced twice for the Westslope cutthroat trout in 2021 and 2022, whereas heart and muscle tissues were sequenced for the same Yellowstone cutthroat trout specimen in 2021. Prior to sequencing, total RNA was extracted using a TRIzol Plus RNA Purification kit (Thermo Fisher Scientific, Waltham, MA, USA), and secondary cleanup was performed using the ProNex Size‐Selective Purification System (Promega, Madison, WI, USA), both according to the manufacturer's instructions. Quantification and qualification of total RNA were evaluated using a Fragment Analyzer RNA kit and Fragment Analyzer (Agilent Technologies, Santa Clara, CA, USA). Iso‐Seq libraries were prepared using a SMRTbell prep kit 3.0 (Pacific Biosciences, Menlo Park, CA, USA) using rRNA depletion, following manufacturer's instructions. Sequencing was completed using a PacBio Sequel II system on four SMRTcells for each tissue with a run‐time of 600 min for each SMRTcell.

### 2.3 Transcriptome assembly

Short‐read Illumina sequences were quality checked with FASTQC (v0.12.1; Andrews, 2010; Table S3) and trimmed using Trim Galore! (v0.6.10; https://github.com/FelixKrueger/TrimGalore). Next, these sequences were assembled using Trinity (v2.8.5; Grabherr et al., 2011) and rnaSPAdes (v3.15.5; Bushmanova et al., 2019). These assemblies were merged using Transfuse (v0.5.0; https://github.com/cboursnell/transfuse) to create a single transcriptome. Finally, we removed duplicate transcripts from our merged assemblies using CD‐HIT‐EST (v4.8.1), a package within CD‐HIT (Fu et al., 2012), to improve the efficiency of downstream analyses.

Long‐read PacBio sequences were quality checked with LongQC (v1.2.1; Fukasawa et al., 2020; Table S4) before being processed according to PacBio's IsoSeq3 (v3.4.0) protocol. Using this protocol, we first generated consensus sequences using ccs (v6.0.0). Next, we demultiplexed the reads using lima (v2.0.0), which removes primers and identifies barcodes within the reads. The full‐length reads were then run through refine (v3.4.0), which identifies and trims poly‐A tails, and removes concatemer reads. Finally, we used cluster (v3.4.0) to generate high‐quality (predicted accuracy ≥0.99) and low‐quality (predicted accuracy <0.99) transcripts by clustering all full‐length non‐concatemer reads together. Going forward, we used high‐quality transcripts for downstream analyses. We removed duplicate transcripts from our assemblies using CD‐HIT‐EST with an 88% similarity threshold. Before we ran CD‐HIT‐EST, we concatenated the assemblies from the different runs of the same tissue for the Westslope cutthroat trout as well as the different tissues for the Yellowstone cutthroat trout.

### 2.4 Transcriptome validation

We generated assembly statistics for the transcriptomes using seqstats (v1.0.0; https://github.com/clwgg/seqstats) and samtools (v1.17; Danecek et al., 2021) throughout the assembly process. We assessed assembly completeness for each transcriptome using BUSCO (Benchmarking Universal Single‐Copy Orthologs; v5.2.2; Simão et al., 2015). BUSCO assesses transcriptome completeness by comparing transcriptomes against precompiled databases of orthologs. For our transcriptomes, we used orthologs found in Actinopterygii, which encompasses all ray‐finned fishes including salmonids. Output included complete BUSCOs (the number of orthologous genes found in the transcripts), single‐copy complete BUSCOs (the number of orthologous genes with only one copy), duplicated complete BUSCOs (the number of orthologous genes that were found more than once), fragmented BUSCOs (the number of orthologous genes partially found), and missing BUSCOs (the number of orthologous genes not found at all). Higher levels of complete BUSCOs indicate higher‐quality transcriptomes.

### 2.5 Ortholog identification

We utilized the single‐copy genes identified by BUSCO for our phylogenetic analyses. Because the amino acid sequences of these genes were nearly, if not entirely, identical between subspecies, we used the nucleotide sequences. The third codon position exhibits the “wobble effect” because it is less functionally constrained and more susceptible to mutations (Bofkin & Goldman, 2007). Theoretically, this wobble can create variation in nucleotide sequences, while maintaining the same amino acid sequence. In addition to the transcriptomes generated for this study, we also included single‐copy genes from three transcriptomes available on NCBI as outgroups: Coho salmon (*Oncorhynchus kisutch*; GDQG00000000.1), Pink salmon (*Oncorhynchus gorbuscha*; GFUW00000000.1), and Rainbow trout (*Oncorhynchus mykiss*; GFIN00000000.1).

We used two filtering steps using CD‐HIT‐EST to confirm that the BUSCO genes used in our analyses were orthologous. In the first step, we used a 95% similarity threshold on only the cutthroat trout transcriptomes to identify BUSCO genes that were dissimilar between the subspecies and excluded these genes from downstream analyses. In the second step, we used an 80% similarity threshold on all transcriptomes, including the outgroup species, to remove any remaining paralogous genes.

### 2.6 Phylogenetic reconstruction

To evaluate the effect of missing data and number of genes in our phylogenies, we generated different sets of orthologous genes using three filtering schemes: low (genes with 13 or more specimens represented), medium (genes with 8 or more specimens represented), and high (genes with 4 or more specimens represented). Using these sets of genes, we generated multiple‐sequence alignments (MSAs) with MAFFT (v7.520; Katoh et al., 2002). We used Clipkit (v2.1.1; Steenwyk et al., 2020) to remove positions in the MSAs that had a gap percentage of ≥80%. To generate concatenated‐based species trees, we concatenated our alignments together using FASconCAT (v1.11; Kück & Meusemann, 2010) and then created a partition definition file using a python script (https://github.com/pbfrandsen/partition_def_from_fcc/tree/main). We identified optimal evolutionary models for each partition in the concatenated supermatrix using ModelFinder (Kalyaanamoorthy et al., 2017) and generated a maximum‐likelihood phylogeny with bootstrap support using IQ‐TREE 2 (v2.2.5; Minh et al., 2020) and UFBoot2 (Hoang et al., 2018).

To generate coalescent‐based species trees, we inferred gene trees using IQ‐TREE 2. Because gene trees can finish in local optima (Nguyen et al., 2015), we ran IQ‐TREE 2 ten times and retained the tree with the highest‐likelihood score (Zhou et al., 2018). Additionally, we modified the perturbation strength (−pers 0.2) and number of stop iterations (−nstop 500; Nguyen et al., 2015). We also generated bootstraps with UFBoot2 and performed SH‐like approximate‐likelihood ratio tests (SH‐like aLRT; Guindon et al., 2010). Prior to inferring a coalescent‐based species tree with local posterior probabilities (LPP) using ASTRAL III (v5.7.1; Zhang et al., 2018), we collapsed gene‐tree branches with bootstraps <30% and with 0% SH‐like aLRT support (Simmons & Gatesy, 2021; Zhang et al., 2018). Additionally, because IQ‐TREE 2 arbitrarily bifurcates polytomies and sets the branch lengths at 0, we collapsed branch lengths that were <1e−8 into polytomies using ape (v5.8) in R (Paradis & Scliep, 2019; R Development Core Team, 2020). We rooted and visualized our phylogenies using FigTree (v1.4.4; https://github.com/rambaut/figtree/) with Pink salmon as the outgroup.

## 3 RESULTS

### 3.1 Transcriptome assembly

All our sequencing runs were of high quality except for the second PacBio run, which had a high percentage of nonsense reads (23.8%). This could be because an additional specimen was sequenced at low coverage, and thus, was not included in future analyses. Before assembling our short‐read sequences, total raw Illumina reads ranged from 25,809,410 to 60,863,364 (Table S1). After assembling with Trinity, the total number of transcripts per specimen ranged from 122,170 to 305,008 transcripts. Our rnaSPAdes assemblies had a range of 122,948 to 265,569 total transcripts, while our combined transcriptomes using Transfuse had a range of 141,491 to 325,290 total transcripts. Finally, we reduced sequence redundancy using CD‐HIT‐EST, resulting in a final set of nine transcriptomes that ranged from 117,148 to 246,508 transcripts (Table S5). Before assembling our long‐read sequences, our first run had 711.4 GB of sequence data with 117,205,232 reads. Our second run had 418.9 GB of sequence data with 89,137,583 reads. After generating circular consensus sequences for our two runs, we had 3,740,383 and 2,415,110 transcripts, respectively. Once we assembled our sequences using IsoSeq3 (demultiplexing, refining, and clustering), our assemblies ranged from 12,789 to 49,593 transcripts per specimen. We further reduced sequence redundancy using CD‐HIT‐EST, resulting in a final set of transcriptomes that ranged from 9177 to 27,529 transcripts (Table S6).

### 3.2 Transcriptome validation

**FIGURE 3:**
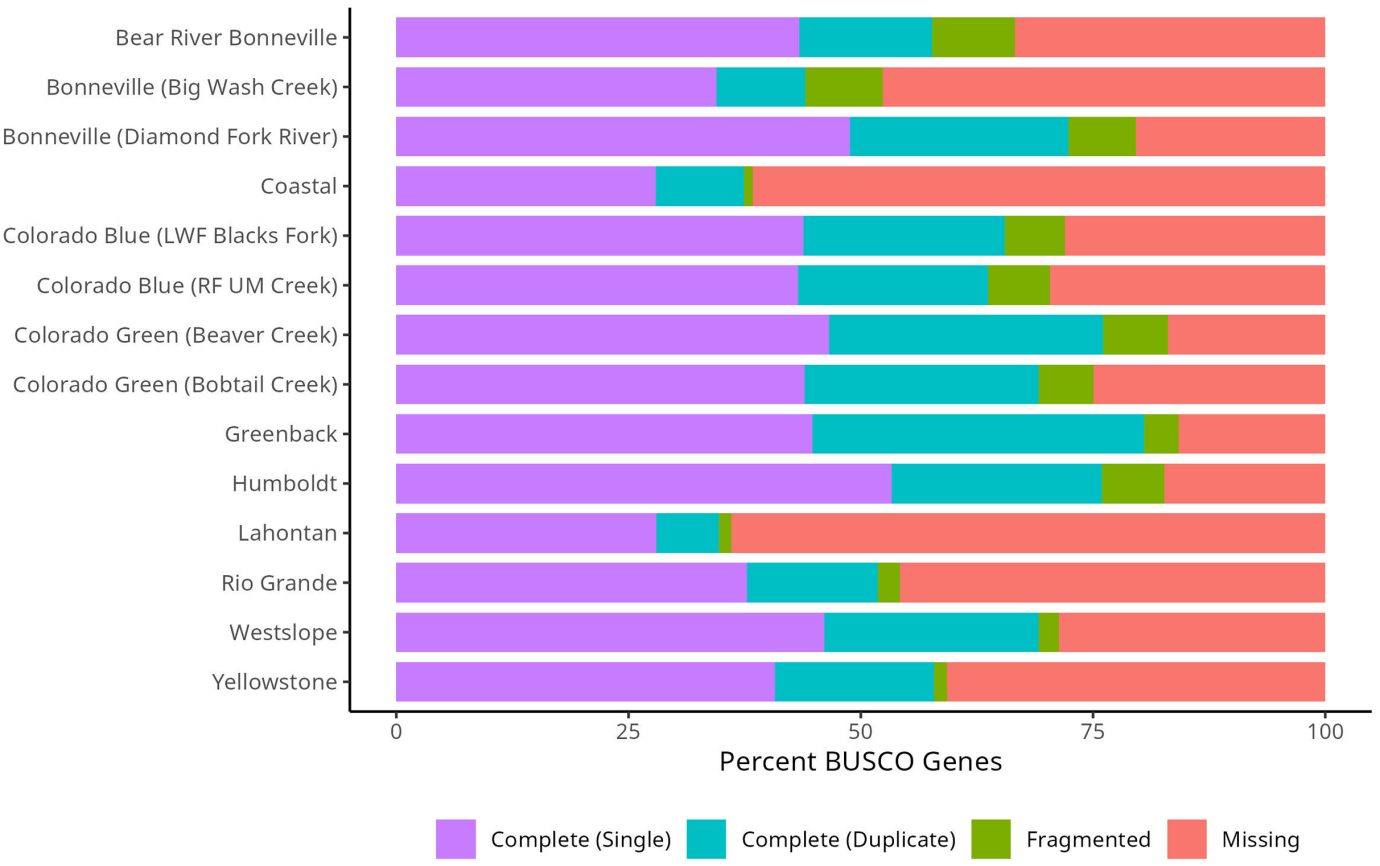
BUSCO gene percentages for each specimen.

We validated the completeness of our transcriptomes with BUSCO. BUSCO indicated that our de novo transcriptomes contained a variety of percentages of complete BUSCOs (highly conserved single‐copy and duplicated orthologs), ranging from 34.8% to 80.5%. Of these complete BUSCOs, we had a range of 27.9%–53.3% single‐copy genes. There was a high number of duplicated orthologs, ranging from 6.8% to 35.7%, which is consistent with the polyploid origin of salmonids. Additionally, the transcriptomes had a range of 15.8%–64% missing BUSCO genes. This could be because of two reasons: (1) low sequence coverage; and (2) sequencing only muscle tissue for the majority of specimens (Figure 3). For example, the Yellowstone cutthroat trout transcriptomes assembled using heart and muscle tissues contained 25.8% and 51.4% complete BUSCOs, respectively, but when combined contained 57.8% complete BUSCOs.

### 3.3 Ortholog identification and phylogenetic reconstruction

**FIGURE 4:**
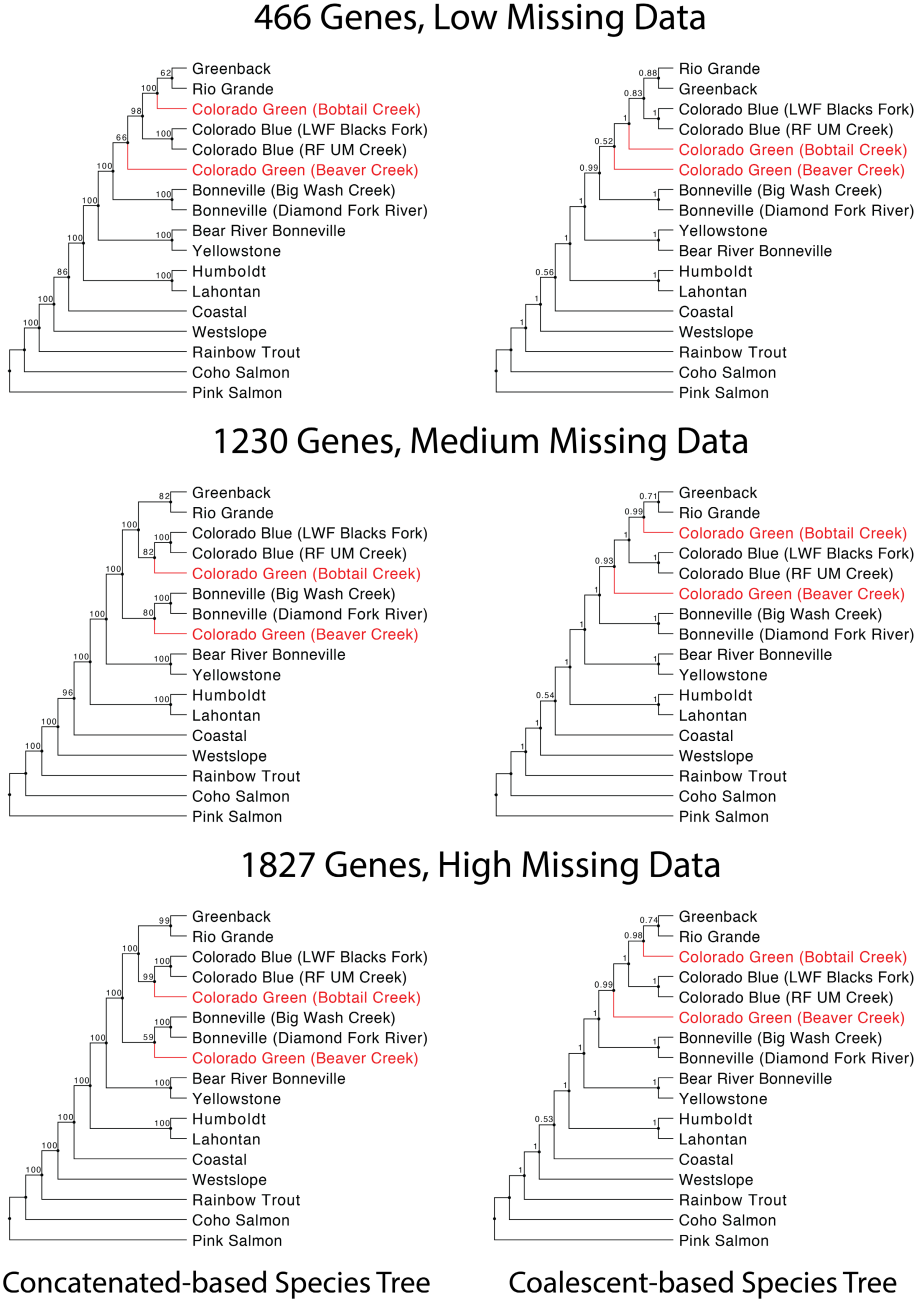
Concatenated‐ and coalescent‐based species trees using three filtering schemes. Support values represented are bootstrap values and local posterior probabilities for each branch for the concatenated and coalescent trees, respectively.

Using three different filtering schemes (low, medium, and high missing data), we identified 623, 1786, and 2892 orthologous genes. After filtering out paralogous genes with CD‐HIT‐EST, 466, 1230, and 1827 genes remained for the gene sets described above, respectively (Table S7). The topology across all phylogenies was the same, except for the placement of the Colorado River green lineage specimens. Specifically, the Colorado River green lineage from Beaver Creek, UT, was either sister to (1) a clade consisting of the Colorado River green lineage from Bobtail Creek, CO, the Colorado River blue lineage specimens, and the Greenback and Rio Grande cutthroat trout, or (2) the Bonneville cutthroat trout. Alternatively, the Colorado River green lineage from Bobtail Creek, CO was sister to three different clades: (1) the Colorado River blue lineage specimens; (2) Greenback and Rio Grande cutthroat trout; and (3) both clades, combined. Many of the relationships in our phylogeny were well supported; however, we consistently identified regions of lower support. The split between Coastal cutthroat trout and the remaining subspecies had bootstrap values ranging from 86% to 100%, while local posterior probabilities (LPPs) ranged from 0.53 to 0.56. For the clade consisting of Greenback and Rio Grande cutthroat trout, the bootstrap values ranged from 62% to 99% and the LPPs ranged from 0.71 to 0.88. Regarding the Colorado green lineage specimens, the bootstrap values and LPPs varied depending on the topology of the phylogeny (Figure 4).

## 4 DISCUSSION

This is the first study to use a large number of nuclear genes to infer the phylogenetic relationships of cutthroat trout subspecies (*O. clarkii ssp*.). Utilizing de novo transcriptomes, we add to previous studies that use karyotypes (Gyllensten et al., 1985), allozymes (Loudenslager & Gall, 1980; Martin et al., 1985), mitochondrial genes (Loxterman & Keeley, 2012; Pritchard et al., 2009; Shiozawa et al., 2010, 2018; Smith et al., 2002; Wilson & Turner, 2009), microsatellites (Pritchard et al., 2007), and SNPs (Campbell et al., 2012; Houston et al., 2012; Kalinowski et al., 2011; Pritchard et al., 2012, 2013). We report the phylogenetic relationships of these subspecies using gene sets extracted from transcriptomes of nine cutthroat trout subspecies as well as one form (Bear River Bonneville) and two lineages (Colorado River blue and green). It is important to note that the completeness of our transcriptomes varied, which led to the introduction of nonrandom missing data in our phylogenetic analyses. Because missing data and the number of genes present can both influence species tree estimation (Nute et al., 2018; Wiens, 2006; Xi et al., 2016), we generated phylogenies using orthologous gene sets identified with different filtering schemes. Despite these limitations, we were able to consistently infer distinct phylogenetic relationships within most of these cutthroat trout subspecies regardless of the amount of missing data or genes in our analyses.

In our phylogenies, Westslope cutthroat trout is sister to all other cutthroat trout subspecies (Figure 4). While Behnke, Shiozawa, and others (Behnke, 2002; Shiozawa et al., 2010, 2018; Smith et al., 2002; Wilson & Turner, 2009) place Coastal as sister to all other subspecies, there have been several studies that are consistent with our placement (Loudenslager & Gall, 1980; Shiozawa & Williams, 1992). This relationship between the Westslope and Coastal cutthroat trout has also been seen in other phylogenies (Allendorf & Leary, 1988; Metcalf et al., 2012; Utter & Allendorf, 1994), although their relationship to the other subspecies differs from our findings.

The relationship between Westslope and Coastal cutthroat trout could have resulted from two different hypothesized invasions, one suggested by Behnke (2002) and the other by Shiozawa et al. (2018). Specifically, Behnke (2002) proposed that starting around 1 mya, cutthroat trout ancestors dispersed from the Columbia River Basin into the interior west of North America, subsequently diversifying into the contemporary subspecies. Alternatively, Shiozawa et al. (2018) proposed an earlier invasion of cutthroat trout through the ancient Lahontan Basin, which would ultimately give rise to the Westslope, Lahontan, and the remaining interior cutthroat trout subspecies (Kimmel, 1975; Shiozawa et al., 2018; Stearley & Smith, 2016). Our phylogeny could be consistent with a combination of these hypotheses, in which there were multiple invasions of ancestral Coastal cutthroat trout into the interior west: (1) an invasion into the Columbia River Basin through the Columbia River, eventually isolating into the Westslope cutthroat trout; and (2) an invasion into the ancestral Lahontan Basin from one or more California coastal rivers as the Great Basin Altiplano collapsed. These trout eventually dispersed further into the interior west as Shiozawa et al. (2018) detailed. However, our coalescent phylogenies had low local posterior probabilities (LPPs) between Coastal cutthroat trout and the remaining subspecies. This could be indicative of the low number of genes represented for Coastal cutthroat trout compared to the other subspecies in our analyses (Table S7). Additional nuclear markers, both coding and non‐coding, along with increased sample representation from other hypothesized lineages of Westslope cutthroat trout, would be helpful to confirm this relationship.

The Yellowstone cutthroat trout and the Bear River Bonneville form comprise a clade in our phylogeny (Yellowstone–Bear River complex), which is sister to the remaining interior cutthroat trout (Southern Rocky Mountain–Bonneville complex). This relationship in the Yellowstone–Bear River complex is consistent with the findings of many studies (Bingham et al., 2016; Gall & Loudenslager, 1981; Houston et al., 2012; Martin et al., 1985; Shiozawa et al., 2018; Shiozawa & Williams, 1992) and contradicts Behnke's (1981) long‐standing hypothesis that Yellowstone cutthroat trout was the ancestral lineage of the interior west cutthroat trout. Instead, our phylogeny suggests the ancestral cutthroat trout line in the ancient Lahontan Basin diverged into these two complexes (Yellowstone–Bear River and the Southern Rocky Mountain–Bonneville).

An invasion into the Upper Snake River could have occurred either directly from the ancient Lahontan Basin, in which cutthroat trout traveled through the upper Humboldt River system and subsequently transferred into the Snake River system through a stream capture; or directly from the Bonneville Basin after cutthroat trout invaded this area from the ancient Lahontan Basin. Possible routes for this latter transfer include a stream capture into the Bear River, or a spillover connection between the Bonneville Basin and Snake River Basin during an early Pleistocene or late Pliocene expansion of Lake Bonneville (Ore et al., 1996; Shiozawa et al., 2018). While the precise invasion route is unknown, we can conclude that once cutthroat trout were established in the Upper Snake River Basin, they had access to or from the Bear River, which was a tributary to the Snake River until ~100–50 kya when lava flows redirected the river into the Bonneville Basin (Bouchard et al., 1998; Bright, 1967; Pederson et al., 2016). This event would have isolated cutthroat trout in the Bear River from those in the Upper Snake River, ultimately resulting in the Bear River Bonneville form. Those in the Upper Snake River became today's Yellowstone cutthroat trout.

It appears that dispersal of cutthroat trout from the Bonneville Basin led to the creation of the remaining members of the Southern Rocky Mountain–Bonneville complex. Populations inhabiting the Bonneville Basin could have dispersed eastward through the Wasatch Mountains or southeastward through the Wasatch Plateau and subsequently entered headwaters in the Colorado River Basin. We were specifically interested in delineating these remaining subspecies in the Southern Rocky Mountain–Bonneville complex because the relationships within this group have remained unresolved (Loudenslager & Gall, 1980; Loxterman & Keeley, 2012; Metcalf et al., 2012; Rogers et al., 2018; Shiozawa et al., 2010, 2018; Smith et al., 2002; Utter & Allendorf, 1994; Wilson & Turner, 2009). Regardless of missing data or number of genes present in our phylogenies, we consistently identified two clades: (1) Colorado River blue lineage (*n* = 2); and (2) the Greenback and Rio Grande cutthroat trout. We found that the Colorado River green lineage specimens were paraphyletic and their placement fluctuated depending on the number of genes used (Figure 4).

The placement of the Colorado River green lineage from Beaver Creek, UT, could be the result of including genes that were not represented in all taxa in our phylogeny (Lemmon et al., 2009) or could be evidence of secondary contact between this specific population and Bonneville cutthroat trout sometime after the lineages in the Colorado River system had been established. This secondary contact could have resulted from human transfer. Records show that several streams in the LaSal mountains were stocked in 1943 with cutthroat trout that originated from the Springville Hatchery in Utah County, UT (Calvin Black, UDWR, personal communication). At that time, their cutthroat trout brood stock may still have consisted of Bonneville cutthroat trout. Dispersal of fish from this stocking event may have reached other streams in the Dolores River Basin, including Beaver Creek of the LaSal Creek drainage.

The monophyletic association between the Greenback cutthroat trout and Rio Grande cutthroat trout suggests that these two lineages on the east slope of the Rocky Mountains came from a single invasion. Since the Yellowfin cutthroat trout of the Arkansas River Basin could not be examined with this technique, we can only infer that it, being geographically located between the South Platte and Rio Grande River Basins, would also fall in this clade. The placement of the Colorado River green lineage from Bobtail Creek, CO in our phylogenies fluctuated depending on the number of genes present (Figure 4). Clearly, additional data are needed to confidently place this specimen within our phylogeny. Depending on where this specimen is placed will provide insight into how the remaining Southern Rocky Mountain–Bonneville complex dispersed. Three of the six phylogenies (low‐threshold concatenated‐based species tree, medium‐ and high‐threshold coalescent‐based species trees; Figure 4) suggest that Greenback and Rio Grande cutthroat trout arose through an invasion of Colorado River green lineage fish, possibly via the Upper Colorado River. However, a separate lineage of Colorado River cutthroat trout has been identified in the San Juan River Basin (Rogers et al., 2018). We were unable to obtain an RNA sample from this lineage but at least two studies (Metcalf et al., 2012; Rogers et al., 2018) suggested a relationship with it and the Colorado River green lineage cutthroat trout. The San Juan cutthroat trout should be examined with whole mtDNA genomes as well as nuclear markers to determine if a southerly path of Colorado River green lineage cutthroat trout, around the Rocky Mountains, is also a viable option for colonization of the eastern slopes of the Rocky Mountains. Alternatively, two of the phylogenies (medium‐ and high‐threshold concatenated‐based species trees; Figure 4) show both Colorado River blue and green lineages as a sister clade to the Greenback and Rio Grande cutthroat trout. This could imply an older invasion event into the eastern drainages of the Rocky Mountains before the western lineages had separated. Finally, one phylogeny (low‐threshold coalescent‐based species tree; Figure 4) suggests that the blue lineage Colorado River cutthroat trout gave rise to the Greenback and Rio Grande cutthroat trout clade.

It has recently been recommended by the American Fisheries Society & American Society of Ichthyologists & Herpetologists (Page et al., 2023) following suggestions by Markle (2018) that the cutthroat trout subspecies be elevated to four species of cutthroat trout: Coastal (*Oncorhynchus clarkii*), Westslope (*Oncorhynchus lewisi*), Lahontan (*Oncorhynchus henshawi*), and Rocky Mountain (*Oncorhynchus virginalis*) cutthroat trout. These recommendations are based on the unified species concept, which defines species as “separately evolving metapopulation lineages” and treats existing criteria, such as reproductive isolation and monophyly, as lines of evidence in favor of a species (De Queiroz, 2007). Based on these recommendations, the Rocky Mountain cutthroat trout would consist of the entire Yellowstone complex of Behnke (2002), which includes at least five extant subspecies. From our findings, it appears that if the recent cutthroat trout species elevations are retained, additional phylogenetically distinct groups within the Rocky Mountain cutthroat trout species likely warrant species‐level consideration based on the species concept utilized above. The elevation of the Yellowstone cutthroat trout (*O. clarkii bouvieri*) would include two subspecies: the Yellowstone cutthroat trout in the Upper Snake River Basin and the Bear River cutthroat trout of the Bear River subbasin in the Bonneville Basin. Despite the transfer of the Bear River into the Bonneville Basin and the subsequent overflow of Lake Bonneville into the Snake River (Bright, 1967; Malde, 1968), molecular evidence shows little gene flow from these events into recipient basins. This is supported by allozyme data beginning in the 1980s (Loudenslager & Gall, 1980; Martin et al., 1985), multiple mitochondrial DNA studies (Loxterman & Keeley, 2012; Shiozawa et al., 2010, 2018), and including this one, three nuclear DNA studies (Bingham et al., 2016; Houston et al., 2012). At this time, we agree with Markle (2018) that a molecular investigation of the Yellowstone cutthroat trout‐type specimen should be undertaken to verify that it represents the Yellowstone cutthroat trout and not the Westslope cutthroat trout. We suggest the common name of the Bear River Bonneville form in the Bear River subbasin to be the Bear River cutthroat trout to recognize that this is not a Bonneville cutthroat trout by descent and to enable ongoing management programs to continue with minimal disruption.

We also recommend recognition that the Bonneville cutthroat trout (*O. clarkii utah*) is the form endemic to the Bonneville Basin proper. This group is on a distinctive, separate phylogenetic trajectory. The name represents its original species designation (Suckley, 1874), which was based on a specimen collected from Utah Lake, the type locality for this subspecies. We also suggest retaining the common name Bonneville cutthroat trout, which was applied to this species from Utah Lake by Jordan (1891); see also Markle (2018). Other authors have designated the cutthroat trout in the Bear River as the Bonneville cutthroat trout and the cutthroat trout in the main Bonneville Basin as the Great Basin cutthroat trout (Campbell et al., 2012; Keeley et al., 2021; Loxterman & Keeley, 2012). These designations are in error for two reasons. First, the type specimen of the Bonneville cutthroat trout is from Utah Lake, which, being a remnant of the Pluvial Lake Bonneville (Brimhall & Merritt, 1981), is clearly in the main Bonneville Basin. Thus, the epithet *utah* is tied to that type specimen and that location with nomenclatural precedence. Second, designating the main Bonneville Basin cutthroat trout as the Great Basin cutthroat trout mis‐states the area encompassed by the Great Basin, which extends from the east slope of the Sierra Nevada and Southern Cascades to the Wasatch Back of Utah and from southern Oregon to Death Valley, CA, encompassing over 520,000 km^2^ (Shiozawa et al., 2023; Shiozawa & Rader, 2005). The largest two endorheic basins, in the northern portion of the Great Basin, the Bonneville Basin (~117,000 km^2^) and the Lahontan Basin (~108,000 km^2^; https://waterdata.usgs.gov/nwis) combined comprise about 43% of the Great Basin. Both have endemic cutthroat trout lineages. The Lahontan Basin is occupied by the Lahontan cutthroat trout. Renaming cutthroat trout in the main Bonneville Basin, the Great Basin cutthroat trout is therefore geographically misleading. We propose that designation of the subspecies common names benefit from more precise geographical nomenclature.

From our phylogeny, it appears that many of the subspecies in the newly designated Rocky Mountain (*O. virginalis*) cutthroat trout species warrant their own species‐level designation. This includes the Yellowstone, Bonneville, Colorado River blue lineage, Greenback, and Rio Grande cutthroat trout. The recent elevation of the cutthroat trout into four species sets the stage for confusion as the diversity in the Rocky Mountain cutthroat trout becomes clearer with time and genomic investigation. Our phylogenies show this diversity as these trout represent monophyletic lineages, often nested within discrete basins. Moving forward, increasing the number of specimens examined for each subspecies as well as adding other subspecies and lineages not represented in this study will provide additional support for the relationships identified in our phylogenies. In addition, other techniques, including those focused on genome‐level analyses, will provide more robust information for understanding the relationships among the cutthroat trout and are needed prior to the formal elevation of these subspecies.

## CONFLICT OF INTEREST STATEMENT

The authors have no conflicts of interest to declare.

## Supporting information


Appendix S1

## Data Availability

Data for this study are openly available at NCBI at https://www.ncbi.nlm.nih.gov/bioproject/PRJNA1006326/. Code for this study is available at Zenodo https://doi.org/10.5281/zenodo.12509951 (Searle, 2024).
