# Supplementary material for: Using de novo transcriptomes to decipher the relationships in cutthroat trout subspecies (Oncorhynchus clarkii)
Source: Evol Appl. 2024 Jul 11;17(7):e13735. doi: 10.1111/eva.13735 (PMC11239772; doi:10.1111/eva.13735)
Supplement: Supplementary file 1 — Appendix S1 [file EVA-17-e13735-s001.docx]

Table S1. Illumina sequencing information

| Specimen | Year | Location | Read Length | Total Sequences |
| --- | --- | --- | --- | --- |
| Bear River Bonneville form | 2015 | BYU, UT | 2x100 | 25809410 |
| Bonneville (Big Wash Cr) | 2022 | Novogene, CA | 2x150 | 40736716 |
| Bonneville (Diamond Fk) | 2015 | BYU, UT | 2x100 | 42262810 |
| Colorado River blue lineage (LWF Blacks Fk) | 2022 | Novogene, CA | 2x150 | 57154706 |
| Colorado River blue lineage (RF UM Cr) | 2022 | Novogene, CA | 2x150 | 57289942 |
| Colorado River green lineage (Beaver Cr) | 2022 | Novogene, CA | 2x150 | 49877108 |
| Colorado River green lineage (Bobtail Cr) | 2022 | Novogene, CA | 2x150 | 60863364 |
| Greenback | 2019 | BYU, UT | 2x250 | 60122490 |
| Humboldt | 2015 | BYU, UT | 2x100 | 37591676 |

Table S2. PacBio sequencing information. The same tissue was sequenced twice for the Westslope cutthroat trout in 2021 and 2022, whereas heart and muscle tissues were sequenced for the same Yellowstone cutthroat trout specimen in 2021.

| Specimen | Run | Year | Location |
| --- | --- | --- | --- |
| Coastal | 1 | 2021 | BYU, UT |
| Lahontan | 1 | 2021 | BYU, UT |
| Rio Grande | 2 | 2022 | BYU, UT |
| Westslope | 1 | 2021 | BYU, UT |
| Westslope | 2 | 2022 | BYU, UT |
| Yellowstone (heart) | 1 | 2021 | BYU, UT |
| Yellowstone (muscle) | 1 | 2021 | BYU, UT |

Table S3. FastQC files for Illumina short-read sequences.

| **Specimens** | **Filenames** |
| --- | --- |
| Bear River Bonneville form | BearRiver_1_fastqc.html  BearRiver_2_fastqc.html |
| Bonneville (Big Wash Cr) | Bonneville2_1_fastqc.html  Bonneville2_2_fastqc.html |
| Bonneville (Diamond Fk) | Bonneville1_1_fastqc.html  Bonneville1_2_fastqc.html |
| Colorado River blue lineage (LWF Blacks Fk) | LWFBlackBCR_1_fastqc.html  LWFBlackBCR_2_fastqc.html |
| Colorado River blue lineage (RF UM Cr) | UMBCR_1_.fastqc.html  UMBCR_2_.fastqc.html |
| Colorado River green lineage (Beaver Cr) | BeaverGCR_1_fastqc.html  BeaverGCR_2_fastqc.html |
| Colorado River green lineage (Bobtail Cr) | BobtailGCR_1_fastqc.html  BobtailGCR_2_fastqc.html |
| Greenback | Greenback_1_fastqc.html  Greenback_2_fastqc.html |
| Humboldt | Humboldt_1_fastqc.html  Humboldt_2_fastqc.html |

Table S4. LongQC files for PacBio long-read sequences. The same tissue was sequenced twice for the Westslope cutthroat trout in 2021 and 2022, whereas heart and muscle tissues were sequenced for the same Yellowstone cutthroat trout specimen in 2021.

| **Run (Year)** | **Specimens** | **Filenames** |
| --- | --- | --- |
| 2021 | Coastal, Lahontan, Westslope, Yellowstone (heart, muscle) | PacBio_run1_2021.html |
| 2022 | Rio Grande, Westslope | PacBio_run2_2022.html |

Table S5. Assembly statistics for Illumina short-read sequences. n represents the total number of transcripts.

| **Specimen** | **Trinity** | | **rnaSPAdes** | | **Transfuse** | | **CD-HIT-EST** | |
| --- | --- | --- | --- | --- | --- | --- | --- | --- |
|  | n | N50 | n | N50 | n | N50 | n | N50 |
| Bear River Bonneville form | 158056 | 1368 | 207571 | 1093 | 231539 | 1415 | 177902 | 1210 |
| Bonneville (Big Wash Cr) | 122170 | 1011 | 122948 | 845 | 141491 | 1135 | 117148 | 901 |
| Bonneville (Diamond Fk) | 219336 | 1542 | 265569 | 1373 | 307318 | 1650 | 228071 | 1505 |
| Colorado River blue lineage (LWF Blacks Fk) | 219859 | 1352 | 192781 | 1501 | 228203 | 1755 | 178021 | 1539 |
| Colorado River blue lineage (RF UM Cr) | 236284 | 1221 | 207949 | 1318 | 246114 | 1590 | 194515 | 1352 |
| Colorado River green lineage (Beaver Cr) | 305008 | 1337 | 261133 | 1470 | 325290 | 1677 | 246508 | 1536 |
| Colorado River green lineage (Bobtail Cr) | 260523 | 1299 | 225370 | 1459 | 270307 | 1701 | 211583 | 1459 |
| Greenback | 230198 | 1724 | 145062 | 2385 | 192436 | 2506 | 144546 | 2430 |
| Humboldt | 198747 | 1599 | 238955 | 1439 | 274593 | 1702 | 202627 | 1596 |

Table S6. Assembly statistics for long-read sequences. Only high-quality transcripts are reported for the clustering step (HQ). The same tissue was sequenced twice for the Westslope cutthroat trout in 2021 and 2022, whereas heart and muscle tissues were sequenced for the same Yellowstone cutthroat trout specimen in 2021. Westslope and Yellowstone samples were combined, respectively, before running CD-HIT-EST. n represents the total number of transcripts. Run 1 and 2 occurred in 2021 and 2022, respectively.

| **Specimen** | **Run** | **Demultiplexed** | | **Refined** | | **Clustered (HQ)** | | **CD-HIT-EST** | |
| --- | --- | --- | --- | --- | --- | --- | --- | --- | --- |
|  |  | n | N50 | n | N50 | n | N50 | n | N50 |
| Coastal | 1 | 1139833 | 5140 | 1138481 | 5111 | 21467 | 3911 | 10489 | 3985 |
| Lahontan | 1 | 431791 | 3350 | 429840 | 3320 | 19384 | 3628 | 9177 | 3731 |
| Rio Grande | 2 | 670380 | 2820 | 669533 | 2793 | 49593 | 2917 | 19976 | 3115 |
| Westslope | 1 | 544400 | 2874 | 540338 | 2841 | 22082 | 3089 | 27529 | 2907 |
| Westslope | 2 | 976425 | 2208 | 975227 | 2180 | 57058 | 2463 |  |  |
| Yellowstone (heart) | 1 | 275188 | 2568 | 274592 | 2539 | 12789 | 2833 | 19953 | 4111 |
| Yellowstone (muscle) | 1 | 956416 | 3541 | 954104 | 3495 | 37804 | 4166 |  |  |

Table S7. Number of orthologous genes represented in each species based on gene sets generated using different thresholds of missing data.

| **Specimen** | **Low** | **Medium** | **High** |
| --- | --- | --- | --- |
| Bear River Bonneville form | 416 | 836 | 988 |
| Bonneville (Big Wash Cr) | 344 | 679 | 802 |
| Bonneville (Diamond Fk) | 425 | 939 | 1146 |
| Coastal | 245 | 453 | 567 |
| Coho salmon | 414 | 1012 | 1365 |
| Colorado River blue lineage (LWF Blacks Fk) | 419 | 938 | 1072 |
| Colorado River blue lineage (RF UM Cr) | 420 | 933 | 1053 |
| Colorado River green lineage (Beaver Cr) | 408 | 877 | 1085 |
| Colorado River green lineage (Bobtail Cr) | 416 | 944 | 1089 |
| Greenback | 405 | 886 | 1095 |
| Humboldt | 435 | 1001 | 1239 |
| Lahontan | 271 | 481 | 587 |
| Pink salmon | 401 | 898 | 1173 |
| Rainbow trout | 432 | 1036 | 1455 |
| Rio Grande | 373 | 751 | 876 |
| Westslope | 415 | 910 | 1091 |
| Yellowstone | 366 | 713 | 885 |
